# A one-dimensional slope detection approach

**DOI:** 10.1186/2193-1801-2-474

**Published:** 2013-09-20

**Authors:** Xiaochun Zhang, Chuancai Liu

**Affiliations:** School of Computer Science and Engineering, Nanjing University of Science and Technology, Nanjing, 210094 China

**Keywords:** Slope, Gaussian, Threshold, Error function, Sub-sample, Localization, Interpolation, Noise suppression, The first derivative operator

## Abstract

This paper extends the scale-invariant edge detector to the one-dimensional slope. It can accurately detect the slope and estimate its parameters. The method has been verified with several mathematical functions, sample sizes, and noise levels. A contrast-invariant operator is proposed to suppress noise. The inter-sample localization and interpolation greatly improve the accuracy. The proposed slope detector is also suitable for real-world signals. In additional to above-mentioned, a threshold formula is developed for the first derivative slope detector, and the upper-bound of the filterable noise level is also explored.

## Introduction

This paper concerns the slope detection of one-dimensional discrete signals (Oppenheim and Willsky 1983). The one-dimensional slope detection shares some common problems (e.g. noise suppression, threshold selection) with the two-dimensional case (Bansal et al. 2012, Pinho and Almeida 2012). This paper is dedicated to these problems.

The edge detection has attracted the attention of many researchers. To suppress signal noise, Witkin proposed a scale space by convolving the input signal with the Gaussian distribution function (Witkin 1983). Canny treated the first derivative of the Gaussian distribution function as one-dimensional edge detector (Canny 1986). Marr et al. studied edge detection using the zero crossing of the second derivative of the Gaussian (Marr 1980). Zhang et al. found a scale-invariant edge detector (Zhang and Liu 2013).

Although two-dimensional edge detection is a well-established subject, however, it seems that the slope of one-dimensional discrete signal is seldom studied. The first derivative operator is a seemingly easy solution. This paper will discuss its problems in detail, and give the remedies. Noise suppression is an important subject of slope detection. Inspired by a contrast-invariant differential operator (Zhang and Liu 2013), a method capable of effectively differentiating noise and slope is proposed. The selection of the smoothing scale is another major problem of many existing methods. The scale-invariant edge detector can address the issue by automatically choosing the adequate scale for each edge feature. This research extends the technique to the one-dimensional slope detection. Because of the apparent distinction between the one dimensional and the two dimensional signals, the scale-invariant edge detector is adjusted for the slope with additional functions of inter-sample localization and interpolation. The proposed method is suitable for several broad classifications of signals: noisy and noiseless, periodic and non-periodic, densely and sparsely sampled.

## One dimensional slope detection

### Signal representation

**Figure 1:**
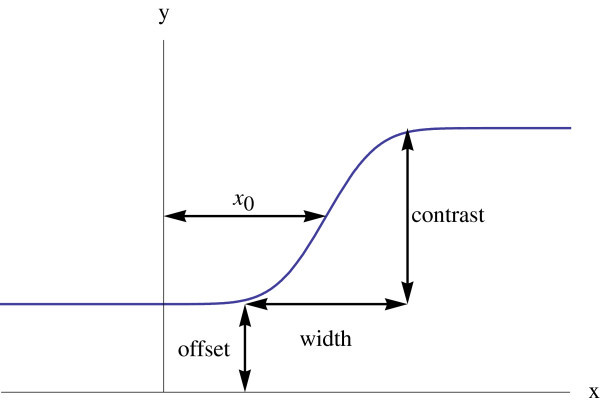
**An ideal slope and its parameters.**

An ideal slope function is needed to investigate detectors quantitatively. The function is characterized by a position, contrast, offset, and width parameters (Figure 1). These parameters can be modeled by the *x*_0_, *c*, *d*, and *w* constant parameters of an error function (Equation 1).1

A step is just a special ideal slope with zero width (Equation 2), therefore all forthcoming discussions are equally applicable.2

For a function *f* having the shape of a slope, two steps are involved to acquire a slope of width *w*, contrast *c*, position *x*_*0*_, and offset *d*, as shown in Equation 3. The min and max functions return minimum and maximum value of their inputs. The discussion is based on the ideal slope (Zhang and Liu 2013), and the result is verified by several slope functions.3

Convolving the input slope function and the Gaussian distribution function results in the scale space (Equation 4) of the input signal where the *σ* represents the scale. It is easy to verify that the *L* function is also an error function.4

The error function has nice properties. For example, with a variable substitute (Equation 6), both the error function and its scale space are solutions of the heat equation:56

Because the scale space of the ideal slope and its differential operators are represented in closed-form, further investigation can be carried out.

### Scale and spatial detector

The *Lx* operator is used in this paper to denote the scale-normalized derivative of *L* with respect to *x* (Equation 7). The scale-normalization is necessary for a scale-invariant detector (Zhang and Liu 2013). The derivative of *Lx* with respect to scale is shown in Equation 8.78

To detect a slope on a specific scale, the operator should have a local extremum along the scale coordinate axis. In order to show that *Lxs* satisfies the requirement, Equation 9 (Zhang and Liu 2013) is used to substitute variables. The *ξ* and *Q* can be treated as scale-normalized *x* and *w*.9

**Figure 2:**
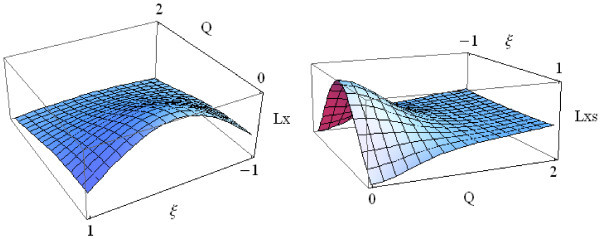
**The transformed** ***Lx*** **and** ***Lxs.***

Figure 2 shows the variable substituted version of *Lx* and *Lxs*. The *Lx* operator has a local extremum along the *ξ* coordinate axis, and the *Lxs* operator has a local extremum along the *Q* coordinate axis.

The location of *Q* is solved by taking the derivative of *Lxs* with respect to *Q* and evaluating to zero (Equation 10).10

**Figure 3:**
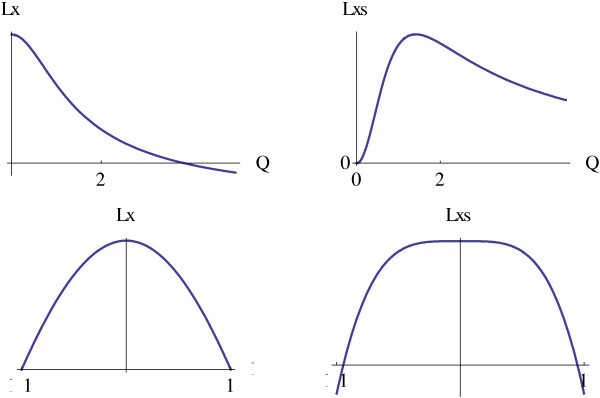
**The spatial and scale perspective of** ***Lx*** **and** ***Lxs*** **.**

The same thing can be illustrated by Figure 3. Using planes *ξ*=0 and *Q*=*sqrt*(2) to intersect *Lx* and *Lxs* results in curves. The diagram in the upper left quadrant of Figure 3 shows *Lx* lacking a local extremum along *Q* coordinate axis. On the other hand, *Lxs* has a local extremum along *Q* coordinate axis (upper right quadrant). From the diagrams in the lower left and lower right quadrants of Figure 3, it seems that both operators having a local extreme along *ξ* coordinate.

To ensure the existence of a local extremum of *Lxs* along the *ξ* coordinate axis, the second derivative of *Lxs* with respect to *ξ* is computed, obtaining a zero (Equation 11).11

The fact indicates that *Lxs* has not a local extremum along the *ξ* coordinate and therefore can not locate spatial position. To detect the position and scale of a slope, two operators are necessary.

### Parameter estimation

The slope is detected when *Lxs* reaches a local extremum along the scale coordinate. As shown in Equation 12, the contrast of the slope can be solved for using *Lxs* and the offset can be solved for using *L* (value at the extremum location). To detect step slopes, a pre-smoothing operation with a small scale is required. The pre-smoothing scale is removed from the detected scale, results in the width.12

### Inter-sample localization and interpolation

Directly applying the method of continuous functions will produce noticeable errors for discrete signals. Sub-pixel localization can be applied to three-dimensional scale space functions (Brown and Lowe 2002, Lowe 2004). This paper adopts a slightly different approach, because the scale and position are detected by two different operators. Taking the derivative of the Taylor expansion of a function can determine the inter-sample location. The *Lxs* operator is used for scale localization (Equation 13) and *Lx* for spatial localization (Equation 14).1314

**Figure 4:**
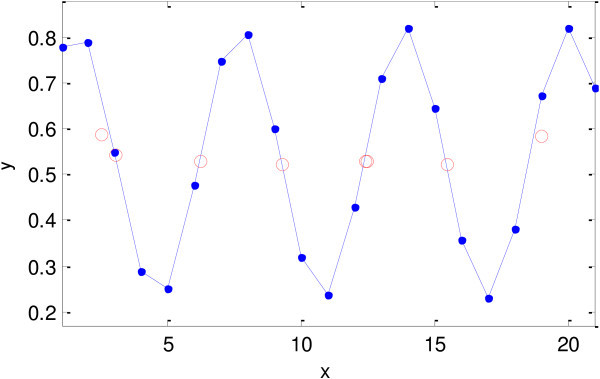
**Sparsely sampled sine slopes detected by the proposed detector.**

Bilinear interpolation using the refined location and scale is involved to compute the parameters. The inter-sample localization and interpolation procedure greatly improves the detection and estimation accuracy. Figure 4 illustrates located slopes between samples and the interpolated vertical position (*d*+*c*/2).

### Implementation

The scale space (*L*) and the *Lx* spaces are computed in the frequency domain (Cooley and Tukey 1965):15

Where *σ*_*s*_ is the discrete smoothing scale given by Equation 20.

The Gaussian and the derivative of the Gaussian convolution kernels can be computed using the expression:16

The *Lxs* is implemented using a central difference:17

The scale and spatial derivatives of *Lx* and *Lxs* can be obtained using differences (Equation 18 and Equation 19), and these values are used for the inter-sample localization.1819

In Table 1 and Equation 20, the maximum scale ensures that the convolution kernel will not exceed the signal boundary. Only slopes with contrast greater than 0.05 are considered therefore low contrast slopes are ignored as a noise. Because narrow slopes (e.g. step) are difficult to detect; it is necessary to smooth the signal first using some small scale. However, for better accuracy, pre-smoothing operation is not performed for the parameter estimation experiment.20

**Table 1:** **Implementation configuration**

Signal samples	***σ***_0_	S	Maximum scale
513	0.6	6	513/6

## Noise suppression

It is well-known that the first derivative operator is extremely sensitive to noise; therefore a smoothing operation is often carried out to suppress noise. Although a large scale can filter the high level noise, signal details are also removed. The smoothing scale is often selected empirically, and a minimum scale is necessary to filter a certain noise level (mean and standard deviation). The minimum scale can only be determined by the scale-invariant detectors (instead of *L*_*x*_). Therefore the following paragraphs deduce the threshold of *L*_*x*_ for a pre-smoothing scale.

### The threshold of the first derivative operator

The first derivative of the smoothed ideal slope at the location of *x*_*0*_ can be obtained using Equation 21, where the *σ* is the pre-smoothing scale.21

If the minimum and maximum values are denoted by subscript *m* and *M*, then the low and high threshold of the *L*_*x*_ detector are represented by *T*_*m*_ and *T*_*M*_ (Equation 22). The *T*_*M*_ and the first component of *T*_*m*_ filter slopes whose contrast is within the range of *c*_*m*_ and *c*_*M*_, and the width within *w*_*m*_ and *w*_*M*_. A noise can be filtered out by the second component of *T*_*m*_, if the standard deviation (*std*^*n*^) and mean (*mean*^*n*^) of the noise are known. It is assumed that the highest contrast of a Gaussian noise is six times the standard deviation plus the mean, and its lowest width is *w*_*m*_^*n*^ (a small value, e.g. 0.5). If the second component of *T*_*m*_ is smaller than the first component, then all slopes within the range are detectable. If the second component of *T*_*m*_ is between the first one and *T*_*M*_, then only slopes of partial range are detectable. Otherwise, no slope can be detected. Besides, six times standard deviation is guaranteed to filter out all noise points in principle, while three times standard deviation may preserve a few noise points.22

In order to detect slope and filter out all noise, the second component of *T*_*m*_ should be no more than *T*_*M*_ (equation 23). An upper bound of the filterable noise is achieved with an infinite large pre-smoothing scale. The noise with higher standard deviation can not be completely removed from the detectable slopes.23

As defined by Table 2, the highest contrast of synthesized ideal slopes is *1*, hence the highest contrast of filterable noise is also *1*. Without loss of generality, the experiments are based on Gaussian noise series of zero mean and random standard deviation. The *L*_*x*_ detector uses the thresholds (*T*_*m*_ and *T*_*M*_) to filter out noise. The errors of *L*_*x*_ are effectively reduced, as shown in Table 3 and Table 4.

**Table 2:** **Parameter ranges for ideal slopes**

Parameter	Minimum ( ***m*** )	Maximum ( ***M*** )
**Width (** ***w*** **)**	1	50
**Contrast (** ***c*** **)**	0.05	1
**Offset (** ***d*** **)**	0.05	1
***x***_***0***_	−40	40

**Table 3:** **RMSD of positional errors of the** ***L***_***x***_ **detector for the noisy ideal slopes, without considering noise threshold**

***std*** ^***n***^	Pre-smooth	Erf	Tanh	Sigmoid	Ramp	Step	Arctan
**0.001**	**1**	92.1	96.31	88.28	94.26	145.67	95.77
	**4**	8.92	8.14	51.64	13.01	0.58	81.91
	**64**	0.3	0.3	0.3	0.41	0.58	0.3
**0.01**	**1**	147.65	148.06	146.24	148.34	150.46	146.63
	**4**	131.81	136.07	119.53	130.57	143.35	123.38
	**64**	0.39	0.41	0.45	0.46	0.61	0.42
**0.1**	**1**	149.72	149.85	149.61	149.77	150.2	149.58
	**4**	147.27	147.78	146.49	147.99	148.9	146.55
	**64**	56.84	60.87	58.98	56.84	52.13	63.43

**Table 4:** **RMSD of positional errors of the** ***L***_***x***_ **detector for the noisy ideal slopes, considering noise threshold**

***std*** ^***n***^	Pre-smooth	Erf	Tanh	Sigmoid	Ramp	Step	Arctan
**0.001**	**1**	34.64	33.4	52.53	16.65	0.58	56.26
	**4**	8.64	4.5	47.17	12.76	0.57	75.2
	**64**	0.28	0.28	0.29	0.4	0.57	0.29
**0.01**	**1**	24.53	21.04	39.43	15.36	0.58	38.86
	**4**	15.08	10.36	33.27	14.11	0.57	38.06
	**64**	0.39	0.41	0.47	0.46	0.58	0.47
**0.1**	**1**	21.34	20.04	47.93	15.17	0.57	51.45
	**4**	10.4	8	28	11.54	0.58	30.67
	**64**	1.05	0.95	1.2	0.86	0.89	1.15

### A method to differentiate slope and noise

Two techniques are adopted by the proposed method to suppress noise. The first is setting contrast threshold (Equation 24), and the second is limiting an operator to some range. The contrast is estimated using equation 12; therefore this sub-section will concentrate on the second technique.24

The ratio of the two operators of the ideal slope is a constant at the extremum location, as shown in Equation 25.25

Table 5 lists the ratios’ range, mean and standard deviation of several slope functions. For each slope function, the experiment uses Table 2 to generate 1000 synthesized slopes. The ratios of different slope functions are similar, because the means are near 2/3, and the standard deviations are small.

**Table 5:** **statistics of** ***Lxs*** **/** ***Lx*** **for various slope functions**

Edge	Min	Max	Mean	Std
**Erf**	0.6381	0.6891	0.6637	0.0149
**tanh**	0.6169	0.9932	0.6488	0.0503
**sigmoid**	0.4503	0.6705	0.6398	0.0193
**ramp**	0.7768	0.8565	0.8134	0.0221
**step**	0.6429	0.6429	0.6429	0
**arctan**	0.5531	0.9968	0.5798	0.0522

**Figure 5:**
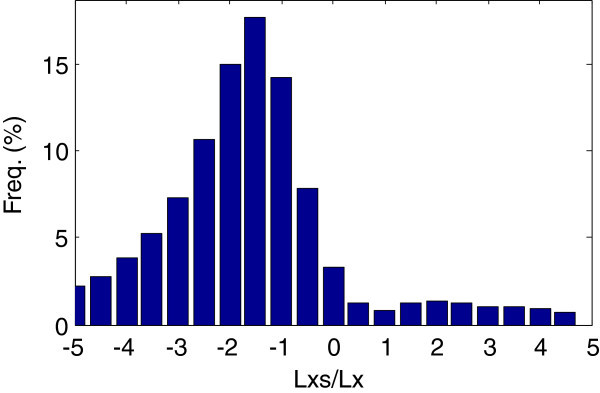
***Lxs*** **/** ***Lx*** **of Gaussian noise of zero mean and random standard deviations, pre-smoothing scale and** ***T***_***m***_ **are 0.**

The ratio behaves differently for noise. Figure 5 illustrates the ratios’ distribution for Gaussian noise. The ratios are mainly located at negative axis, and reach a peak near −1.5.

The following content tries to explain the phenomenon. A noise (impulse) of contrast, offset, and width (*c*, *d*, and *w*) is modeled by:26

As before, the scale space *L* is the Gaussian smoothed signal, and the *Lxs* and *Lx* operators can be obtained from Equation 7 and Equation 8. The ratio equals −2 when both *Q* and *ξ* are zero (Equation 27).27

**Figure 6:**
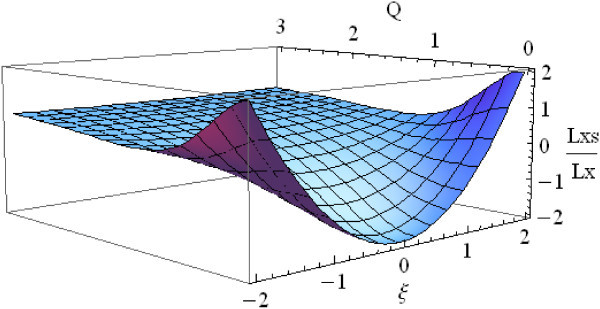
***Lxs*** **/** ***Lx*** **of noise impulse signal.**

The division of *Lxs* and *Lx* is shown in Figure 6. Because the width of the noise impulse is near zero, the location (*x*) of the feature is also near zero. And because the smoothing scale is not zero, according to Equation 9, *Q* and *ξ* are also near zero. However, since both *Q* and *ξ* are actually small values, the ratio will be slightly larger than −2 (accord with −1.5 in Figure 5).

Experimenting on Gaussian noise series of zero mean (*mean*^*n*^) and random standard deviations (*std*^*n*^), Table 6 lists the percentages of *Lxs*/*Lx* at three intervals under two conditions. The first condition is four pre-smoothing scales, and the second is the contrast thresholds, which is 0, 1, 3, or 6 times standard deviation of the noise. The table indicates that, increasing the contrast threshold will reduce the percentage of *Lxs*/*Lx* at interval [0, 1). The characteristic contributes to noise suppression, because the noise and the slopes are indistinguishable in this interval. Noise is suppressed by large pre-smoothing scale, leading to a lower contrast threshold. The noise is completely removed by pre-smoothing scale *1* and contrast threshold of 6 times standard deviation. The experiment shows that the noise can be suppressed by the following procedures: pre-smoothing the signal with a small scale, setting appropriate contrast threshold, and limiting the *Lxs*/*Lx*.

**Table 6:** **The percentages of** ***Lxs*** **/** ***Lx*** **of noise at three intervals under two conditions**

		***Lxs***/***Lx***	***T***_***m***_			
			0	1×***std***_***n***_+***mean***_***n***_	3×***std***_***n***_+***mean***_***n***_	6×***std***_***n***_+***mean***_***n***_
**Pre-smoothing scale**	**0**	(−∞,0)	81.907	82.916	84.421	89.578
		[0,1)	3.204	0.974	0.222	0.005
		[1,∞)	14.889	16.109	15.357	10.417
	**1**	(−∞,0)	69.709	70.955	80.155	84.211
		[0,1)	11.758	7.465	0.379	0
		[1,∞)	18.533	21.580	19.467	15.790
	**10**	(−∞,0)	28.724	75.932	-	-
		[0,1)	60.152	0.641	-	-
		[1,∞)	11.125	23.427	-	-
	**100**	(−∞,0)	0.149	-	-	-
		[0,1)	97.879	-	-	-
		[1,∞)	1.972	-	-	-

## Experimental

### Quantitative experiments

The quantitative experiments involve 1000 randomly generated synthesized slopes with parameters listed in Table 2. Signal is sampled from −256 to 256 discretely and the slope is centered at *x*_0_.

The positional error (*e*_*x*_) was measured by the distances between the slope center and the detected location (Equation 28). The slope centers could be located between the samples. Except the width of the step slopes, the parameter estimation accuracies have been measured by the relative error where the true value of a quantity is *q* and the inferred value *q*_0_:28

The root mean squared deviation (RMSD) of the error is given by29

where *e*^*n*^ is the *n*th actual error and *ϵ*^*n*^ is assumed to be zero.

The step slopes are pre-smoothed with scale *1* and the width is measured by absolute errors. Except the erf and step slope, the widths are transformed by median values for different function. The estimated parameters of the ideal slopes are more precise than other slope functions (Table 7). Although the signals are sampled at discrete points, the inter-sample locations are precisely recovered. The offset is estimated using the scale space and the estimated contrast, therefore has a slightly lower accuracy.

**Table 7:** **RMSD of positional errors of the proposed method, pre-smoothing scale 0**

Error	Erf	Tanh	Sigmoid	Ramp	Step	Arctan
**Position (point)**	0.0097	0.0140	0.1107	0.2065	0.3527	0.0062
**Width (%)**	1.1900	2.0600	19.2500	6.9900	0.9900	1.3400
**Contrast (%)**	2.6300	7.2600	9.0000	16.4400	7.4300	24.8700
**Offset (%)**	3.7300	10.3800	12.7100	23.4800	22.9800	35.7400

The following experiments use the RMSD of the positional error to evaluate the performances. Except ramp slope, *L*_*x*_ produces similar errors for several slope functions (Table 8). The *L*_*x*_ of ramp is a constant; therefore a local extremum can not be found at the slope center. It can be verified that if *L*_*x*_ is implemented by a central difference, then a pre-smoothing is required for step slopes.

**Table 8:** **RMSD of positional errors of the** ***L***_***x***_ **detector for the ideal slopes**

Pre-smooth	Erf	Tanh	Sigmoid	Ramp	Step	Arctan
**0**	0.2859	0.2859	0.2861	16.9743	-	0.2859
**1**	0.2859	0.2859	0.2861	14.3276	0.5836	0.2859
**2**	0.2859	0.2859	0.2861	10.8449	0.5857	0.2860
**4**	0.2859	0.2859	0.2861	4.2151	0.5558	0.2860

The proposed method achieves lower error because of the inter-sample localization (Table 9). A pre-smoothing is also required for the step slopes, because zero width can not be detected in the scale space.

**Table 9:** **RMSD of positional errors of the proposed detector for the ideal slopes**

Pre-smooth	Erf	Tanh	Sigmoid	Ramp	Step	Arctan
**0**	0.0092	0.0157	0.1024	0.2116	-	0.0686
**1**	0.0067	0.0094	0.1024	0.2068	0.3706	0.0682
**2**	0.0040	0.0049	0.1024	0.2094	0.2950	0.0681
**4**	0.0018	0.0021	0.1052	0.2065	0.2913	0.0683

The following experiments adopt zero-mean Gaussian noise. For the experiment of Table 10, the pre-smoothing scale is 2, and the standard deviation of the noise is a random variable between 0.001 and 1. A lower error is achieved with either a high contrast threshold (*T*_*m*_) or a constraint of *Lxs*/*Lx*.

**Table 10:** **RMSD of positional error of the proposed method for noisy ideal slopes under two conditions**

***T***_***m***_	***Lxs***/ ***Lx*** ∈ [− ***∞*** , ***∞*** )	***Lxs***/ ***Lx*** ∈ [0, 1)
***c***_***m***_	146.69	147.31
**max(** ***c***_***m***_ **,1×** ***std***_***n***_ **)**	145.85	145.16
**max(** ***c***_***m***_ **,3×** ***std***_***n***_ **)**	142.47	33.22
**max(** ***c***_***m***_ **,6×** ***std***_***n***_ **)**	34.87	2.60

Assuming zero noise (omitting the second component of the lower threshold in Equation 22 and Equation 24), the *L*_*x*_ (Table 3) will be more sensitive to noise than the proposed method (Table 11). Even for low level noise, *L*_*x*_ needs large pre-smoothing scales to suppress noise. Both methods sacrifice for high level noise.

**Table 11:** **RMSD of positional errors of the proposed detector for the noisy ideal slopes, without considering noise threshold**

***std*** ^***n***^	Pre-smooth	Erf	Tanh	Sigmoid	Ramp	Step	Arctan
**0.001**	**1**	0.06	0.05	0.13	0.22	0.37	0.08
	**4**	0.06	0.04	0.14	0.21	0.29	0.07
	**64**	0.32	0.28	0.55	0.31	0.35	0.58
**0.01**	**1**	7.29	6.1	8.95	6.16	0.35	6.72
	**4**	0.52	0.43	0.61	0.66	0.3	0.48
	**64**	0.41	0.41	0.65	0.43	0.46	0.73
**0.1**	**1**	116.58	119.78	111.97	117.69	135.31	111.44
	**4**	121.03	123.27	109.43	124.5	128.4	109.58
	**64**	12.12	12.37	10.2	9.64	11.72	13.96

Incorporating noise information to the lower threshold, both methods produce significantly lower errors (Table 4 and Table 12). The proposed method achieves low errors for noisy slopes without large pre-smoothing scale. For a large pre-smoothing scale (e.g. 64), the two methods show similar performances.

**Table 12:** **RMSD of positional errors of the proposed detector for the noisy ideal slopes, considering noise threshold**

***std*** ^***n***^	Pre-smooth	Erf	Tanh	Sigmoid	Ramp	Step	Arctan
**0.001**	**1**	0.06	0.05	0.15	0.23	0.36	0.08
	**4**	0.06	0.05	0.15	0.21	0.29	0.08
	**64**	0.32	0.29	0.55	0.31	0.35	0.58
**0.01**	**1**	2.91	3.15	4.3	4.55	0.35	2.45
	**4**	0.55	0.5	0.68	0.77	0.29	0.48
	**64**	0.44	0.43	0.66	0.41	0.43	0.67
**0.1**	**1**	3.16	2.3	3.94	4.86	0.34	2.32
	**4**	1.58	1.46	2.19	2.09	0.35	1.49
	**64**	1.05	1.04	1.28	0.93	0.92	1.32

In additional to densely sampled signals, the method presented here can also detect slopes of sparsely sampled signals. For these slopes (sample size is 21), the proposed method (Table 13) outperforms *L*_*x*_(Table 14). However the result of sparsely sampled slopes (Table 13) is not as good as that of densely sampled slopes (Table 9).

**Table 13:** **RMSD of positional errors of the proposed detector for the sparsely sampled ideal slopes**

Pre-smooth	Erf	Tanh	Sigmoid	Ramp	Step	Arctan
**0**	0.1004	0.0615	0.2323	0.2547	-	0.0692
**1**	0.0946	0.0484	0.2421	0.2353	0.3519	0.0665
**4**	0.3529	0.2818	0.7248	0.2840	0.2932	0.4723

**Table 14:** **RMSD of positional errors of the** ***L***_***x***_ **detector for the sparsely sampled ideal slopes**

Pre-smooth	Erf	Tanh	Sigmoid	Ramp	Step	Arctan
**0**	0.2926	0.2926	0.2924	2.1233	-	0.2926
**1**	0.2926	0.2926	0.2924	0.3994	0.5738	0.2926
**4**	0.3206	0.3034	0.4762	0.4131	0.5649	0.3446

### Some qualitative examples

**Figure 7:**
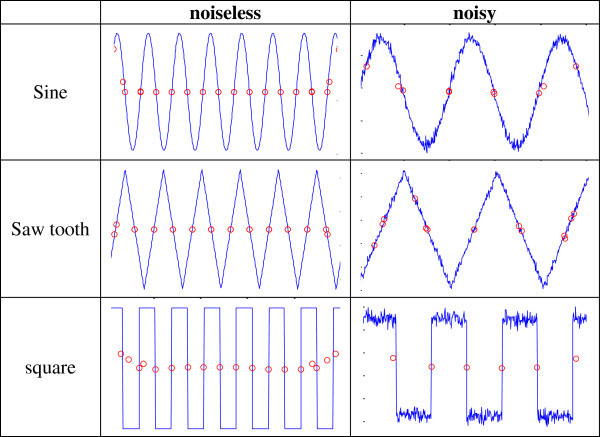
**Slopes of periodic signals.**

A few slopes of periodic signals are displayed in Figure 7, where the signal-to-noise ratio of the noisy signals is 25dB.

**Figure 8:**
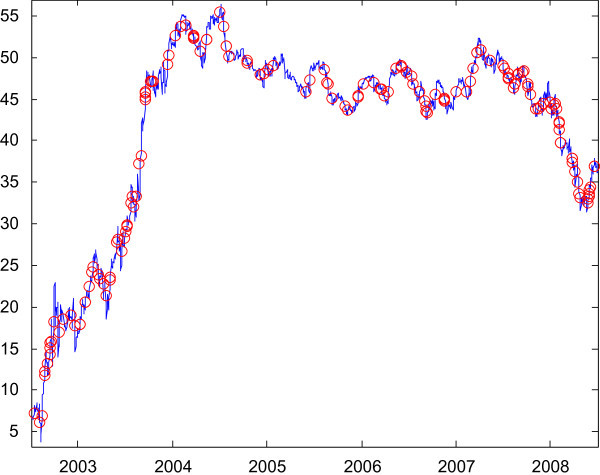
**Slopes detected for stock data by the proposed method.**

Using a script (Renfree 2008), historical stock data can be retrieved from a financial website (Yahoo! Finance 2013). Figure 8 shows historical stock data of CitiBank from January, 2003 to December, 2008, with threshold 1. The proposed method successfully detects slopes of various widths, contrasts, offset,and locations.

## Conclusion

Simple methods such as the first derivative or zero crossings of the second derivative are very sensitive to noise. To detect a slope, two operators are necessary, which should have a local extremum along either the scale or the spatial coordinate axis. The proposed detector involves the scale derivative of the spatial derivative operator, with scale-normalization. Using an error function as a test slope, the parameters are solved for precisely in closed form. A precise inter-sample localization and interpolation procedure is proposed to improve the accuracy. The method can extract slopes from synthesized or real-world signals while detecting less noise than its counterpart methods. Based on mathematical functions, the threshold selection of the first derivative is also discussed.
